# Construction and validation of a machine learning model for the diagnosis of juvenile idiopathic arthritis based on fecal microbiota

**DOI:** 10.3389/fcimb.2024.1371371

**Published:** 2024-03-08

**Authors:** Jun-Bo Tu, Wei-Jie Liao, Si-Ping Long, Meng-Pan Li, Xing-Hua Gao

**Affiliations:** ^1^ Department of Orthopaedics, Xinfeng County People’s Hospital, Xinfeng, Jiangxi, China; ^2^ Department of ICU, GanZhou People’s Hospital, GanZhou, Jiangxi, China; ^3^ The First Clinical Medical College of Nanchang University, Nanchang, China; ^4^ Department of Orthopedics, Shanghai General Hospital, Shanghai Jiao Tong University School of Medicine, Shanghai, China; ^5^ Department of Orthopaedics, Guangzhou First People’s Hospital, South China University of Technology, Guangzhou, China

**Keywords:** juvenile idiopathic arthritis, fecal microbes, machine learning, diagnosis, XGB algorithm

## Abstract

**Purpose:**

Human gut microbiota has been shown to be significantly associated with various inflammatory diseases. Therefore, this study aimed to develop an excellent auxiliary tool for the diagnosis of juvenile idiopathic arthritis (JIA) based on fecal microbial biomarkers.

**Method:**

The fecal metagenomic sequencing data associated with JIA were extracted from NCBI, and the sequencing data were transformed into the relative abundance of microorganisms by professional data cleaning (KneadData, Trimmomatic and Bowtie2) and comparison software (Kraken2 and Bracken). After that, the fecal microbes with high abundance were extracted for subsequent analysis. The extracted fecal microbes were further screened by least absolute shrinkage and selection operator (LASSO) regression, and the selected fecal microbe biomarkers were used for model training. In this study, we constructed six different machine learning (ML) models, and then selected the best model for constructing a JIA diagnostic tool by comparing the performance of the models based on a combined consideration of area under receiver operating characteristic curve (AUC), accuracy, specificity, F1 score, calibration curves and clinical decision curves. In addition, to further explain the model, Permutation Importance analysis and Shapley Additive Explanations (SHAP) were performed to understand the contribution of each biomarker in the prediction process.

**Result:**

A total of 231 individuals were included in this study, including 203 JIA patients and Non-JIA individuals. In the analysis of diversity at the genus level, the alpha diversity represented by Shannon value was not significantly different between the two groups, while the belt diversity was slightly different. After selection by LASSO regression, 10 fecal microbe biomarkers were selected for model training. By comparing six different models, the XGB model showed the best performance, which average AUC, accuracy and F1 score were 0.976, 0.914 and 0.952, respectively, thus being used to construct the final JIA diagnosis model.

**Conclusion:**

A JIA diagnosis model based on XGB algorithm was constructed with excellent performance, which may assist physicians in early detection of JIA patients and improve the prognosis of JIA patients.

## Introduction

The gut microbiota plays a crucial role in immune system development and regulation. Autoimmune diseases, marked by the immune system’s attack on healthy cells, lead to inflammation and tissue damage. Dysbiosis in the gut microbiome, such as abnormal enrichment of certain symbionts, diversity loss, or pathogen invasion, has been shown to cause various human diseases. For example, Zaky et al. have identified the role of the gut microbiome in diabetes and obesity-related kidney diseases (Zaky et al., 2021). And several studies have found that gut microbiota disorder is linked to the activity of rheumatic diseases (Yu et al., 2021; Bao et al., 2020).

Juvenile Idiopathic Arthritis (JIA), the most common chronic rheumatic disease in children, is marked by its mysterious origins and sustained arthritis for over six weeks in individuals under 16 years old. The disease exhibits a varied incidence rate, estimated between 1.6 and 23 cases, and a prevalence ranging from 3.8 to 400 per 100,000 children (Gibiino et al., 2018; Weiss, 2022). It often severely impacts the physical and mental health of children, restricting growth and causing joint deformities, thus diminishing the quality of life and social participation (Haverman et al., 2012). Early diagnosis and treatment are critical to improving outcomes and preventing deformities. Clinical symptoms and imaging findings are helpful in the diagnosis of JIA. However, the etiology of JIA remains elusive and inflammatory findings are not always evident as early symptoms, which may delay the diagnosis of JIA and further aggravate the progression of the disease. By identifying particular signs of chronic inflammation, imaging studies are essential to the early diagnosis of JIA. They are also beneficial in tracking the illness and assessing the efficacy of treatment. Nevertheless, this approach is still in its infancy (Stevens and Rudd, 2013; Tsujioka et al., 2023). Previous research indicated that the diagnosis of JIA often necessitates referrals to three different physicians, with an average median time of three months for a definitive diagnosis, indicating that the diagnosis of JIA is currently difficult (Aoust et al., 2017). Therefore, it is of great significance to develop a tool that can accurately diagnose JIA.

With the emergence of digital health and gene sequencing, artificial intelligence (AI) has shown a broad prospect in medical field (Kim et al., 2021). At present, the emergence of electronic medical records (EMR) and the expansion of databases present significant opportunities for ML application in the medical field. Additionally, ML algorithms are frequently employed for prediction of clinical outcomes, tailored treatment, and early illness diagnosis (Goecks et al., 2020; Huang et al., 2018). For instance, Liu et al. had developed efficient machine learning (ML) models for predicting metastatic bone tumors (Liu WC. et al., 2021). Similarly, Li et al. had designed a ML model to predict the incidence of pulmonary infections following spinal cord injuries (Li et al., 2023). With the advent of software for quality control and precise alignment of metagenomic sequencing data (such as kneaddata, bracken, Kracken, etc.) (Wood et al., 2019; Lu et al., 2022), our understanding of gut microbiota has become more accurate and in-depth, and it is also possible for gut microbiota to be used as predictors for the construction of machine learning prediction models. For example, Su et al. used species based on fecal microbial species level to construct a machine learning predictive model for the prediction of multiple diseases (Su et al., 2022).

In previous studies, the relationship between fecal microbiome and JIA had been explored (Tejesvi et al., 2016). Many studies have pointed out that the pathophysiology of JIA is linked to the gut microbiome (De Filippo et al., 2019; van Dijkhuizen et al., 2019; Qian et al., 2020). A study by Tejesvi et al. (2016) found that the fecal microbiota in JIA presents a high level of Bacteroidetes and a low level of Firmicutes, and changes in the gut microbial ecology may put genetically predisposed individuals’ mucosal immune systems at risk, which could lead to local proinflammatory cascades and the development of JIA. However, fecal microbiome-based ML diagnostic models for JIA are rare. Therefore, in this study, we aimed to integrate phylum and genus-level gut biomarkers to construct and validate a high-performance ML model for assisting JIA diagnosis.

## Methods

### Metagenomic datasets

**Figure 1:**
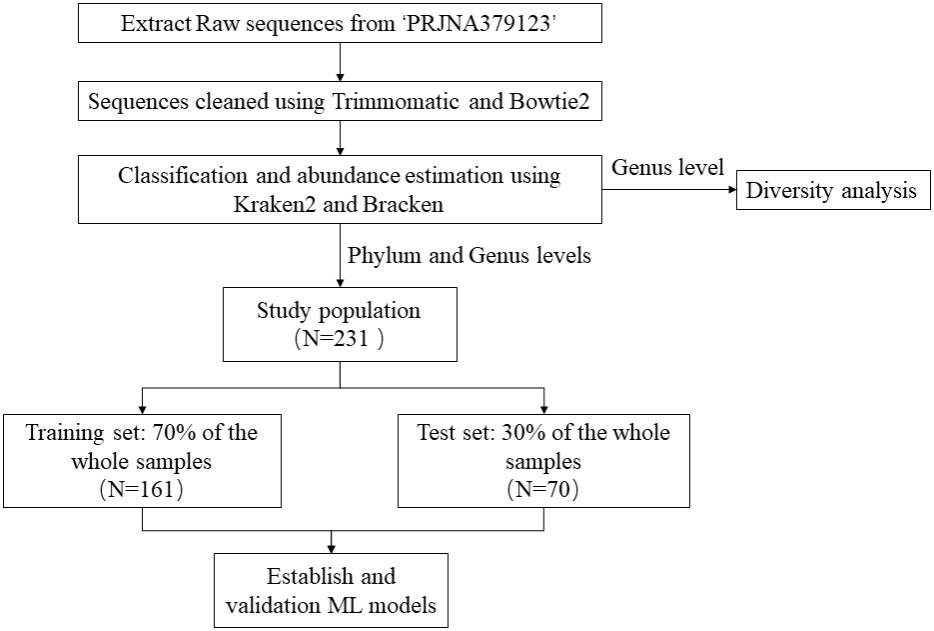
The flow chart of the study.

The metagenomic data utilized in this study were derived from the NCBI project PRJNA379123. We downloaded the FASTQ files of 16S rRNA gene sequences extracted from the fecal samples. Metagenomic data from the experimental group were exclusively derived from fecal samples collected from juvenile idiopathic arthritis (JIA) patients at the initial treatment phase. **Figure 1** illustrated the research flow of this study.

### Sequencing data processing and microbiome profiling

Firstly, we used the KneadData tool to clean and control the raw FASTQ file. The quality of all reads was managed with Trimmomatic (version 0.39), with parameters set to SLIDINGWINDOW:4:20 MINLEN:50 LEADING:3 TRAILING:3 (Bolger et al., 2014). Reads containing human sequences were filtered out using Bowtie2 (version 2.4.5), applying the human reference database (hg37_and_human_contamination) recommended by KneadData, with parameters configured to –very-sensitive –dovetail (Langmead and Salzberg, 2012).

Subsequently, the cleansed FASTQ data were compared against sequences from known microbes with the goal of translating metagenomic 16S rRNA sequencing data into species abundance information. The metagenomic data were classified using Kraken software version 2.2.1.3, with reference to the official Kraken2/Bracken 16S RNA indexes (Silva 138) (Wood et al., 2019; Lu et al., 2022). For precise quantification of microbial abundance as determined by Kraken2, Bracken version 2.9 was employed (Lu et al., 2017). The read counts were converted into relative abundances of gut microbiota at both the phylum and genus levels through Bracken software for subsequent analysis.

### Microbiome analysis and screening

Microbiome analysis and screening and statistical analysis were performed using Python 3.8 and R version 4.3.2. Descriptive statistics were assessed using chi-square tests or Fisher’s exact tests as appropriate. Continuous variables were compared using Student’s t-tests or rank-sum tests. P-value of less than 0.05 considered statistically significant.

To understand the distribution of gut microbiota in the study population, we performed diversity analysis at the genus level, including α-diversity and β-diversity, based on the data after kraken2 classification and bracken abundance estimation. Alpha diversity is often used to measure the number of species in a single sample or environment (richness) and how evenly these species are distributed (evenness). We calculated the Shannon’s α-diversity index (Sh) for each sample using the alpha_diversity.py script from KrakenTools. However, beta diversity is often used to measure the differences in species composition across environments or regions. In this study, β-diversity was examined using Principal Coordinates Analysis (PCoA) based on the Bray–Curtis distance matrix, which was computed using the relative abundances of microbial genus. This facilitated the visualization of sample clustering according to their genus-level compositional profiles. Differences in microbiome composition among various phenotypes were determined using permutational multivariate analysis of variance (PERMANOVA) with distance matrices (adonis) via the adonis function of the vegan R package v.2.6-4.

To reduce the risk of overfitting the prediction model, we need to screen suitable variables before training the model. Initially, we selected the top three phyla and the top twenty genera ranked by average abundance to reduce the influence of technical error on the results. Subsequently, to refine the variables used to train the ML model, these 23 variables were further filtered by least absolute shrinkage and selection operator (Lasso) regression. Features with nonzero regression coefficients in the LASSO model were chosen to train the subsequent ML predictive models.

### Model establishment and evaluation

In this study, all data were randomly divided into training and test sets in a 7:3 ratio. The Synthetic Minority Over-sampling Technique (SMOTE) method was used to oversampling the training set to mitigate the potential impact of imbalanced data on model training (Solihah et al., 2020; Wu et al., 2019). The secret to this approach is to oversample the small class data samples in order to increase the number of small class data samples and boost the model’s accuracy. To identify the most effective ML model for diagnosing juvenile idiopathic arthritis, we trained six commonly used ML models, including three ensemble algorithms and three simple classification algorithms: Random Forest (RF), eXtreme Gradient Boosting (XGB) and Gradient Boosting Machine (GBM) are ensemble algorithms. Naive Bayes Classifiers (NBC), Decision Tree (DT) and Logistic Regression (LR) are three simple classification algorithms. In model construction, each model underwent internal ten-fold cross-validation and tuned hyperparameters. Subsequently, ROC curves and calibration curves for each model were plotted in both the training and test sets to comprehensively assess model performance, aiming to select the model with optimal efficacy for the diagnosis of juvenile idiopathic arthritis. Additionally, to visually demonstrate the net benefit of each model at varying clinical decision thresholds, clinical decision curves were plotted for the models in both training and test sets. Ultimately, the best-performing model for disease diagnosis was selected based on a combined consideration of AUC value, accuracy, specificity, F1 score, calibration curves, and clinical decision curves.

### Feature importance analysis and model demonstration

Shapley Additive Explanations (SHAP) and Permutation Importance analysis are frequently utilized for elucidating ML models (Altmann et al., 2010; Li et al., 2022). The presentation of feature importance not only aids in interpreting the predictive process of ML models but also substantially contributes to our understanding of the roles various microbiota play in the onset and progression of diseases. Through a randomization of the feature test data values and measuring the average error they introducing into the model, permutation importance determines which features are more accurate for a trained model. Different from permutation importance, the SHAP computes each feature’s contribution to the predicted value in order to identify the feature’s significance (Goings and Hammes-Schiffer, 2020; Liu LP. et al., 2021). Therefore, both methods were used to explain the prediction models in this study. In addition, for a more transparent demonstration, we conducted SHAP value visualization by randomly selecting samples from both the experimental and control groups. This approach distinctly illustrates the contribution of different features to the final prediction value when the model predicts outcomes for individual samples.

## Results

### Basic characteristics of the dataset

The present investigation sourced its dataset from the NCBI project PRJNA379123, submitted by the Bambino Gesù Children’s Hospital, IRCCS, Rome, Italy, incorporating a cohort of 231 European individuals. This dataset spans across four distinct phenotypes: three stages of JIA—baseline, inactive, and persistent activity—and a cohort of healthy controls. Our analysis consolidates all JIA conditions into a unified experimental group to delineate the association between the gut microbiota and JIA. Consequently, the study designates 203 individuals as the experimental group and 28 as the control group, with the intent to construct ML models for JIA diagnosis.

### Microbiome analysis and biomarkers screening

**Figure 2:**
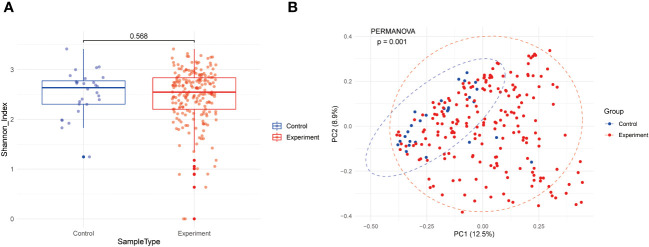
Genus-level diversity of fecal microorganisms. **(A)** alpha diversity; **(B)** belt diversity.

Following metagenomic data processing, including cleaning, taxonomic classification, and abundance estimation, our study performed a diversity analysis of gut microbiota at the genus level. As shown in **Figure 2A**, alpha diversity was evaluated by Shannon index, and there was no significant difference between the JIA group and the healthy group (mean values were 2.47 and 2.52, respectively, P=0.568). However, Principal Component Analysis (PCA) in **Figure 2B** indicated subtle distinctions in beta diversity between JIA patients and healthy individuals (P=0.001).

**Figure 3:**
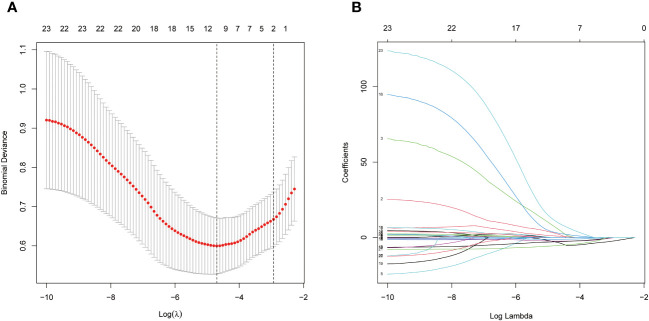
Through LASSO binary logistic regression analysis, ten fecal microbe biomarkers were selected, 3 phylum-level biomarkers (Firmicutes, Bacteroidota and Proteobacteria) and 7 genus-level biomarkers (Faecalibacterium, Alloprevotella, UCG-002, Dialister, Lachnoclostridium, Monoglobus and Veillonella). **(A)** Penalty maps of the Lasso model for 23 biomarkers; **(B)** LASSO coefficient mapping of 23 biomarkers.

To train a high-performance diagnostic model, we initially selected biomarkers based on the top three phyla and top twenty genera by average abundance, with their distribution across the experimental and control groups presented in **Table 1**. At the phylum level, Firmicutes dominate the gut microbial distribution in this population, with a higher relative abundance in healthy individuals than in JIA patients. Bacteroidota and Proteobacteria followed, with higher prevalence in the JIA group. In addition, at the genus level, only Faecalibacterium, with a mean relative abundance exceeding 0.1, showed no significant difference between the groups. To prevent overfitting due to an excess of biomarkers, a LASSO regression was applied to the 23 preselected biomarkers, culminating in the identification of 10 variables for the subsequent model construction and validation, including 3 phylum-level biomarkers (Firmicutes, Bacteroidota and Proteobacteria) and 7 genus-level biomarkers (Faecalibacterium, Alloprevotella, UCG-002, Dialister, Lachnoclostridium, Monoglobus and Veillonella) (**Figure 3**).

**Table 1:** Summary descriptives table by groups of `juvenile idiopathic arthritis’.

Gut microflora	Control	Experiment	*P*-value
	*N=28*	*N=203*	
Phylum level			
Firmicutes_1672	0.91 (0.11)	0.66 (0.26)	<0.001
Bacteroidota_43868	0.05 (0.09)	0.17 (0.18)	<0.001
Proteobacteria_2375	0.01 (0.01)	0.12 (0.19)	<0.001
Genus level			
Faecalibacterium_45544	0.22 (0.19)	0.13 (0.14)	0.023
Bacteroides_43874	0.02 (0.06)	0.07 (0.11)	0.001
Subdoligranulum_45553	0.07 (0.06)	0.06 (0.10)	0.477
Escherichia.Shigella_46463	0.00 (0.01)	0.04 (0.09)	<0.001
Alloprevotella_43941	0.01 (0.01)	0.04 (0.08)	<0.001
Ruminococcus_45552	0.05 (0.08)	0.03 (0.08)	0.144
Blautia_45422	0.04 (0.05)	0.03 (0.05)	0.414
Streptococcus_1853	0.02 (0.03)	0.03 (0.07)	0.188
UCG.002_45530	0.07 (0.08)	0.02 (0.07)	0.009
Dialister_45783	0.06 (0.13)	0.02 (0.06)	0.131
Bacillus_1688	0.03 (0.05)	0.02 (0.06)	0.430
Christensenellaceae.R.7.group_45329	0.03 (0.04)	0.02 (0.07)	0.296
Lachnoclostridium_45446	0.00 (0.00)	0.02 (0.09)	0.001
Pseudomonas_3723	0.00 (0.00)	0.02 (0.08)	0.001
uncultured_43978	0.01 (0.03)	0.02 (0.06)	0.113
Alistipes_43965	0.01 (0.01)	0.02 (0.05)	0.005
Flavobacterium_44221	0.01 (0.02)	0.01 (0.05)	0.152
Monoglobus_45507	0.03 (0.06)	0.01 (0.03)	0.177
Akkermansia_46831	0.01 (0.01)	0.01 (0.05)	0.048
Veillonella_45786	0.00 (0.01)	0.01 (0.04)	<0.001

### Model selection and performance evaluation

**Figure 4:**
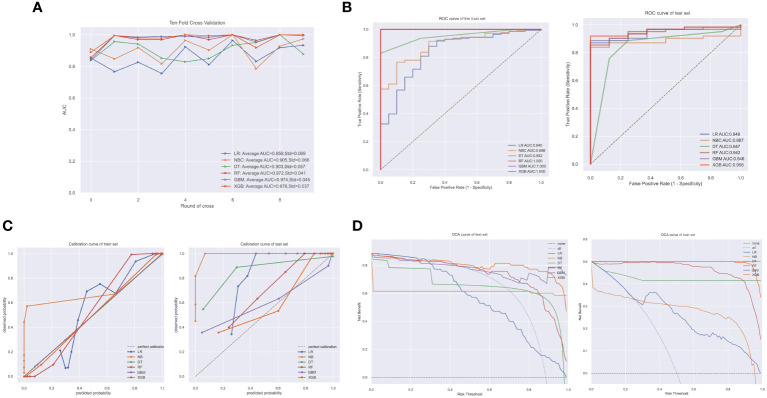
Demonstration of model performance. **(A)** ten-fold cross-validation results of different machine learning (ML) models in training dataset; **(B)** ROC curves of different ML models in training set and test set; **(C)** calibration curves of different ML models in training set and test set; **(D)** decision curve analysis (DCA) of different ML models in training set and test set.

**Figure 5:**
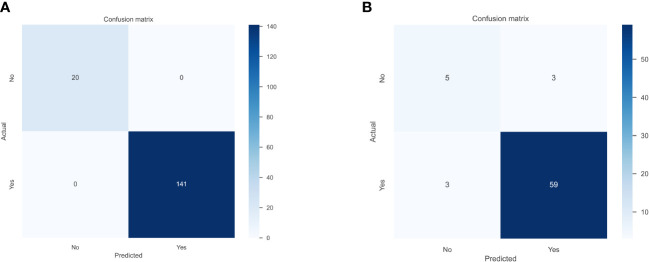
Confusion matrix of the diagnostic model constructed by the XGB algorithm. **(A)** training set; **(B)** test set.

After screening the variables, we trained six ML models based the ten biomarkers. In internal ten-fold cross-validation, the XGBoost (XGB) model emerged as the most effective, achieving an average AUC of 0.976 (**Figure 4A**). The ROC curves for both training and test sets underscored the XGB model’s exceptional performance (**Figure 4B**). Calibration curves for each model further substantiated the XGB model’s accuracy and interpretability, showcasing a closer alignment with perfect calibration in both datasets (**Figure 4C**). Other performance metrics such as accuracy, AUC, Recall, precision, and F1 index of the six models in the test set are detailed in **Table 2**, where the RF, GBC, and XGB models demonstrated remarkable effectiveness. Clinical decision curve analysis confirmed the XGB model’s superior net benefit across nearly all risk thresholds, especially in the test dataset (**Figure 4D**). Considering the overall performance, the XGB model was selected as the diagnostic tool for JIA. **Figure 5** presented the confusion matrix of the final diagnostic model in the training and test sets.

**Table 2:** Performance metrics of different models.

Models	Accuracy	AUC	Recall	Precision	F1
LR	0.700	0.948	0.661	1.000	0.796
NB	0.671	0.887	0.629	1.000	0.772
DT	0.771	0.847	0.758	0.979	0.855
RF	0.900	0.942	0.903	0.983	0.941
GBM	0.886	0.946	0.919	0.950	0.934
XGB	0.914	0.958	0.952	0.952	0.952

RF, Random Forest; XGB, eXtreme Gradient Boosting; GBM, Gradient Boosting Machine; NBC, Naive Bayes Classifiers; DT, Decision Tree; LR, Logistic Regression.

### Feature importance analysis and prediction process presentation

**Figure 6:**
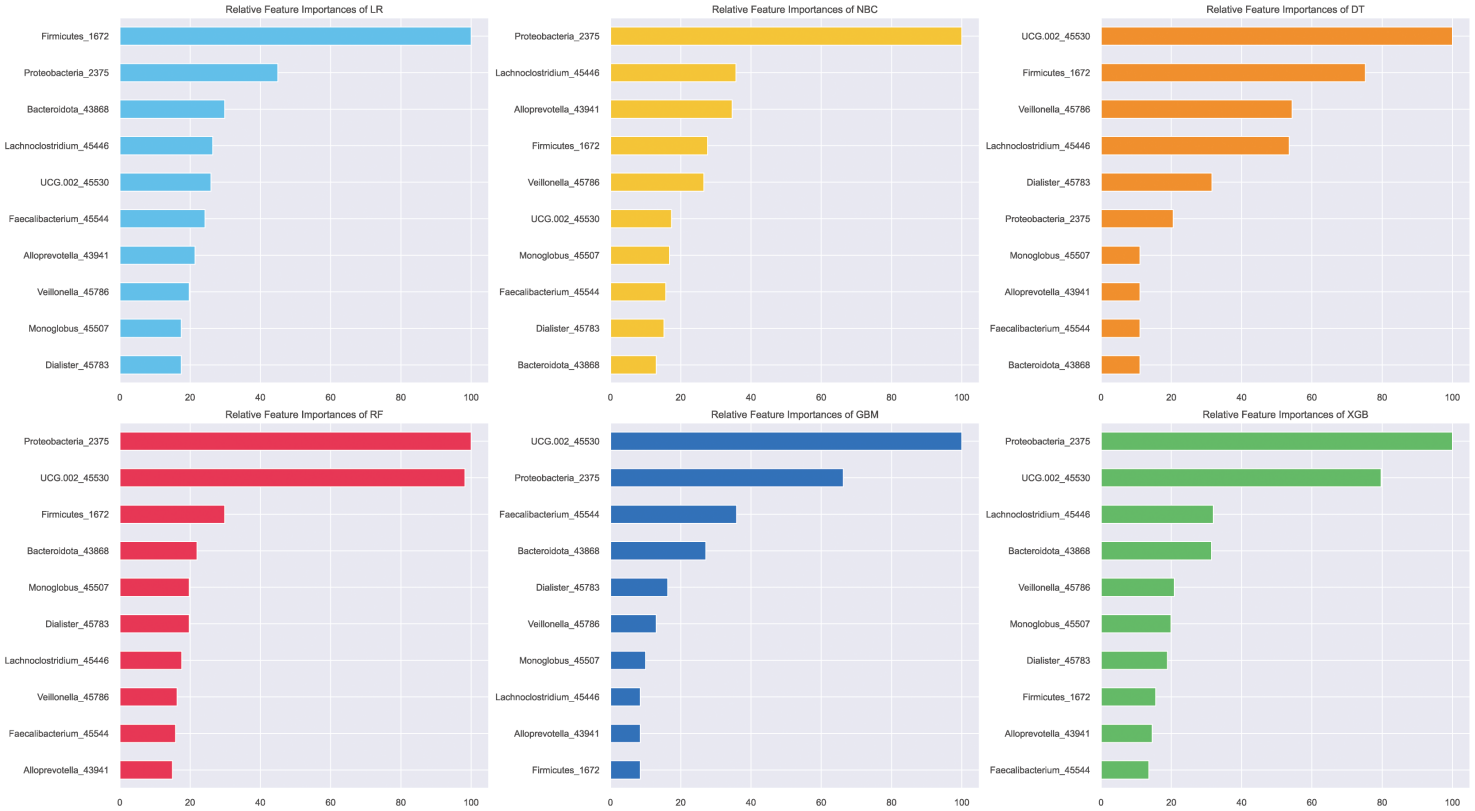
Permutation importance analysis of different models.

**Figure 7:**
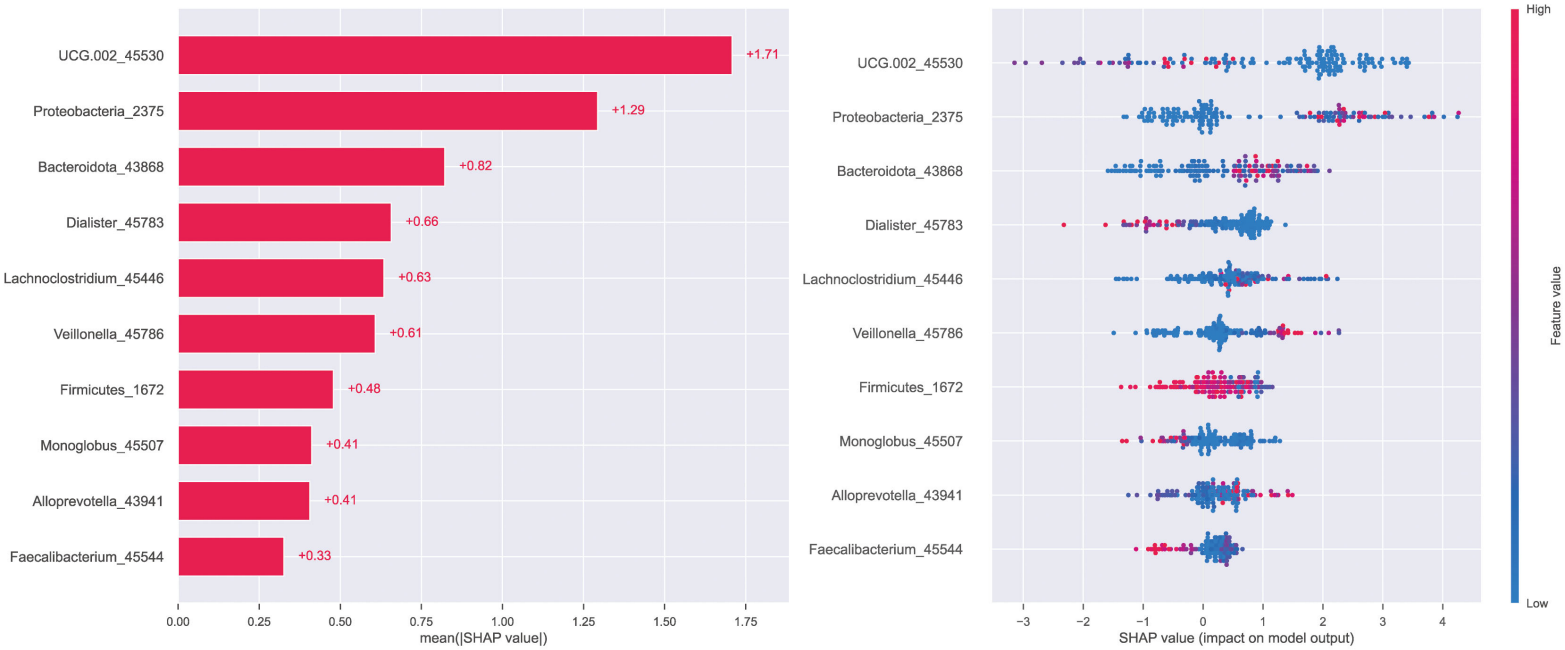
SHAP features analysis of the XGB model.

To explain the role of different biomarkers in the predictive mechanism, we performed a feature importance analysis. Initially, a permutation feature importance assessment across all six models highlighted that Proteobacteria and genus UCG-002 provided the most substantial contribution within the top-performing models—RF, GBC, and XGB (**Figure 6**). The subsequent SHAP analysis of the XGB model also yielded the same result that the contributions of these two biomarkers were significantly higher than those of other biomarkers, followed by Bacteroidota, among others (**Figure 7**).

**Figure 8:**
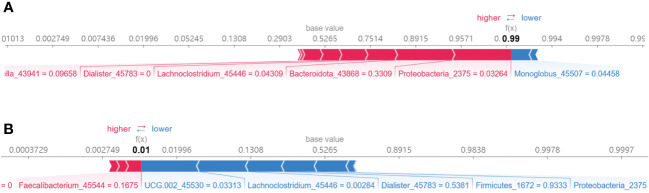
Demonstration of the prediction process of the XGB model. **(A)** A JIA sample; **(B)** A health sample.

In addition, **Figure 8** presented the diagnostic model’s analytic process through SHAP value visualization. **Figure 8A** specifically demonstrates the model’s predictive sequence for a JIA sample, with an outcome of f(x) = 0.99, suggesting a high likelihood of JIA as assessed by the diagnostic model. The numbers following the biomarkers detailed their individual contributions to the prediction. **Figure 8B** showed the prediction process of the model for a healthy sample.

## Discussion

Juvenile Idiopathic Arthritis (JIA) is a relatively uncommon disease that not only affects joints but can also involve other organs. The limited understanding of JIA among pediatricians and general practitioners, coupled with the absence of characteristic symptoms, leads to a high incidence of misdiagnosis, missed diagnosis, and delayed diagnosis. A retrospective study from France, analyzing the diagnostic journey of 67 JIA patients, highlighted these challenges (Aoust et al., 2017). The study revealed that prior to a confirmed diagnosis of JIA, patients had consulted with an average of three physicians, and the median time to diagnosis was 3 months, underscoring the significant difficulties encountered in accurately diagnosing JIA. The most common initial misdiagnoses were Reactive Arthritis (34%) and Septic Arthritis (24%) (Aoust et al., 2017). The treatment approaches for these conditions differ markedly from JIA, and misdiagnosis resulting in prolonged antibiotic use not only hinders recovery but may also promote the development of JIA by disrupting the balance of the human microbiome (Horton et al., 2015). Therefore, the development of a simple and effective tool for diagnosing JIA is of great significance.

Artificial intelligence (AI) is a broad field that enables computers to mimic human intelligence to perform tasks, including understanding language, recognizing images, solving scientific problems, and learning (Laskaris, 2015). Machine Learning (ML), a subset of AI, focuses on developing algorithms and techniques that allow computers to learn from data and make decisions or predictions (Alhusain et al., 2013). ML algorithms achieve learning by analyzing and identifying patterns in data (Alhusain et al., 2013). Considering the abundance of data accessible online and the emergence of electronic medical records (EMR), more clinical data sets, including clinical diagnoses and laboratory data, could be obtained conveniently, thus making ML bring a bright future in medical filed (Deo, 2015; Handelman et al., 2018; Toh et al., 2019; Bhavsar et al., 2021).

In this study, we innovatively constructed six different ML classification models based on fecal microbiomics, including the conventional logistic regression model and ensemble ML models commonly used in the medical field, such as RF, GBM and XGB. Ultimately, XGB model was chosen to construct the diagnostic tool for JIA. Previously, the RF model was commonly employed for processing fecal microbiomics data, and it was generally considered more suitable for handling such data. For instance, Huang et al. developed an RF model based on fecal microbiomics data to predict tumor patients’ responses to PD-L1 antibodies (Huang et al., 2023), and Su et al. also constructed an RF model for multi-disease classification using fecal microbiome data (Su et al., 2022). In our study, an RF model was also developed in the pre-construction phase of the models, which demonstrated excellent performance across various evaluation metrics. However, compared to the XGB model, the RF model was slightly inferior in all aspects, particularly in the calibration curve and clinical decision curve in the test set. This indicates that the XGB model may has stronger generalizability and can bring greater benefits to clinical diagnosis. The XGB algorithm is a scalable, adaptable and effective ML algorithm classifier that has been applied extensively in the medical field, such chronic kidney disease, COVID-19, and bone metastasis (BM) in non-small cell lung cancer (Ogunleye and Wang, 2020; Guan et al., 2021; Li et al., 2022). Li et al. compared six commonly machine learning algorithms and found the XGB algorithm performed best, thus building a web predictor of BM from non-small cell lung cancer (Li et al., 2022). The XGB algorithm included a regular term in the objective function in order to prevent overfitting and manage model complexity. Additionally, it supported column sampling to improve model stability. This could be contributing to the fact that it performed the best in this study (Ester et al., 2022).

In this study, the diversity assessments were conducted at the genus level, which might introduce some deviations compared to the species level. This limitation was due to sequencing quality issues, which prevented accurate extraction of relative abundance of species at the species level (Caporaso et al., 2011; Kuczynski et al., 2011). In addition, the abundance of gut microbiota at phylum level and genus level were extracted to further analyze the influence of gut microbiota on JIA. At the phylum level, a significant reduction in Firmicutes was observed in JIA patients compared to healthy individuals. Firmicutes play a crucial role in immune regulation, as elucidated in the literature. Clarke et al. had explored the relationship between Firmicutes and the immune system, revealing that the gut can process and release glycoconjugates from Firmicutes, promoting cytokine IL-34 release (Jordan et al., 2023). This cytokine stimulates macrophage proliferation, enhancing the body’s defense mechanisms. Additionally, IL-34-mediated Mtorc1 activation in sentinel cells can remove glycoconjugates in peripheral tissues, maintaining immune homeostasis. Our findings indicated a significantly lower proportion of Firmicutes in JIA patients, potentially linked to decreased immune regulation functions.

In evaluating model feature importance and SHAP analysis, we focused on two significant biomarkers: Bacteroidetes and UCG-002. We observed a notably higher relative abundance of Bacteroidetes in the JIA group compared to healthy individuals, with SHAP analysis indicating a positive impact of this biomarker on predicting JIA. As the largest phylum of Gram-negative bacteria found in our guts, Bacteroidetes are regarded as crucial participants in maintaining the complex and healthy homeostasis. It has been proved that several Bacteroidetes genera are linked to the emergence of immunological dysregulation, neurological problems, and systemic diseases including metabolic syndrome (Gibiino et al., 2018). The abundance of the proteobacteria phylum is significantly increased in patients with moderate-to-severe COPD, especially in those with exacerbation of the disease (Pragman et al., 2012). In inflammatory bowel disease, this group of bacteria was also significantly increased (Sartor, 2008). This suggests that the Proteobacteria are important for inflammation promotion, but the underlying mechanisms remain unclear (Rizzatti et al., 2017). UCG-002, belonging to the Ruminococcaceae family, is a key indicator in gut microbiome studies. Lee et al. showed that the high relative abundance of Ruminococcaceae UCG-002 is associated with IgE-mediated food allergy in children (Lee et al., 2021), and Rhee et al. also suggested that Ruminococcaceae UCG-002 genus is a potential factor for psychiatric disorders such as bipolar disorder and major depression (Rhee et al., 2020). Our research found that UCG-002 contributed significantly to JIA, but the underlying mechanisms remain unclear.

Although this study constructed a ML model for JIA diagnosis based on feces with excellent performance, there were still some limitations. First, only sequencing data from a single center were used in this study. In future studies, multi-center data including different ethnic groups are needed for further training of the model to increase the generalization ability of the model. Second, because the data came from a public database, some common confounding factors such as age and gender that may affect the onset of JIA could not be excluded. Third, since species-level relative abundance could not be extracted, only species at the genus and phylum levels were analyzed in this study, and more subdivided species may be more beneficial to construct prediction models with excellent performance in future studies.

## Conclusion

In this study, based on the relative abundance of 10 fecal biomarkers, we used XGB algorithm to construct a JIA diagnosis model with excellent performance, which can assist physicians in early detection of JIA patients and improve the prognosis of JIA patients.

## Data availability statement

The datasets presented in this study can be found in online repositories. The names of the repository/repositories and accession number(s) can be found in the article/supplementary material.

## Ethics statement

This study was approved by the Ethics Committee of Guangzhou First People’s Hospital.

## Author contributions

J-BT: Conceptualization, Investigation, Project administration, Writing – original draft, Writing – review & editing. W-JL: Conceptualization, Data curation, Investigation, Writing – review & editing. S-PL: Conceptualization, Formal analysis, Supervision, Writing – review & editing. M-PL: Conceptualization, Data curation, Formal analysis, Funding acquisition, Investigation, Methodology, Project administration, Resources, Software, Supervision, Validation, Visualization, Writing – original draft, Writing – review & editing. X-HG: Data curation, Funding acquisition, Investigation, Resources, Validation, Writing – original draft, Writing – review & editing.
